# Association of common variants identified by recent genome-wide association studies with obesity in Chinese children: a case-control study

**DOI:** 10.1186/s12881-016-0268-4

**Published:** 2016-01-22

**Authors:** Hai-Jun Wang, Anke Hinney, Jie-Yun Song, André Scherag, Xiang-Rui Meng, Harald Grallert, Thomas Illig, Johannes Hebebrand, Yan Wang, Jun Ma

**Affiliations:** Institute of Child and Adolescent Health, Peking University, Beijing, China; Department of Child and Adolescent Psychiatry, Psychosomatics and Psychotherapy, University Hospital Essen, University of Duisburg-Essen, Essen, Germany; Clinical Epidemiology, Integrated Research and Treatment Center, Center for Sepsis Control and Care (CSCC), Jena University Hospital, Jena, 07743 Germany; Research Unit of Molecular Epidemiology, Institute of Epidemiology II, Helmholtz Zentrum München – German Research Center for Environmental Health, Neuherberg, Germany; German Center for Diabetes Research, Neuherberg, Germany; Hannover Unified Biobank, Hannover Medical School, Hannover, Germany; Division of Maternal and Child Health, School of Public Health, Peking University Health Science Center, Beijing, China

**Keywords:** Obesity, BMI, Gene, Variant, Children

## Abstract

**Background:**

Large-scale genome-wide association studies have identified multiple genetic variants that are associated with elevated body mass index (BMI) or the risk of obesity in Caucasian or Asian populations. We examined whether these variants are individually associated with obesity in Chinese children, and also assessed their cumulative effects and predictive value for obesity risk in Chinese children.

**Methods:**

We genotyped 40 single nucleotide polymorphisms (SNPs) and conducted association analyses for 32/40 SNPs with an estimated minor allele frequency >1 % in 2 030 unrelated Chinese children, including 607 normal-weight, 718 overweight, and 705 obese individuals from two cross-sectional study groups. Logistic regression and linear regression under the additive model were used to examine associations, and the area under the receiver operating characteristic curve (AUC_ROC_) was reported as prediction summary.

**Results:**

We identified obesity association for 6 SNPs near *SEC16B, RBJ, CDKAL1, TFAP2B, MAP2K5* and *FTO* (odds ratios (ORs) ranged from 1.19 to 1.41, nominal two-sided *P*-values < 0.05). Association (Bonferroni corrected) of rs543874 near *SEC16B* and rs2241423 near *MAP2K5* had presumably stronger effects on obesity in Chinese children than in Caucasian populations. Their risk alleles were also associated with BMI standard deviation score (BMI-SDS) variability. We demonstrated the cumulative effects of the 32 SNPs on obesity risk (per risk allele: OR = 1.06, 95 % CI: 1.03-1.11, *P* = 4.84 × 10^-4^) and BMI-SDS (β = 0.04, 95 % CI: 0.02-0.06, *P* = 3.69 × 10^-7^). The difference in AUC_ROC_ for a model with covariates (age, age square, sex and study group) and the model including covariates and all 32 SNPs was 2.8 % (*P* = 0.0002).

**Conclusion:**

While six SNPs were individually associated with obesity in Chinese children, the 32 common variants identified by recent GWA studies had cumulative effects and resulted in a limited increase in the AUC_ROC_ predictive value for childhood obesity.

**Electronic supplementary material:**

The online version of this article (doi:10.1186/s12881-016-0268-4) contains supplementary material, which is available to authorized users.

## Background

The rapid increase of obesity prevalence has been a major public health challenge in both developed and developing countries. Obesity is a major risk factor for many common chronic diseases such as type 2 diabetes mellitus, cardiovascular disease, and many forms of cancer [1]. Although the reason for the increase in obesity prevalence has been largely attributed to environmental factors, including changes in dietary patterns and lifestyle, genetic factors play an important role in obesity susceptibility [2]. The heritability of the variance of body mass index (BMI) ranged from 40 % to 70 % [3].

In 2010, a meta-analysis of genome-wide association (GWA) studies for BMI was conducted in 249,796 adult individuals of European ancestry by the Genetic Investigation of Anthropometric Traits (GIANT) consortium. They confirmed 14 known obesity susceptibility loci and identified 18 new loci associated with BMI at a genome-wide significance level (*P* < 5 × 10^-8^) [4].

Heritability estimations for BMI or obesity are higher in children, compared with adults [5]. Although the currently known major common variants related to obesity overlap to a substantial degree between children and adults, a GWA study in French and German populations identified 2 new loci for childhood obesity including rs121458332 in *SDCCAG8* and rs13278851 near *TNK*S/MSRA. The latter locus had an effect in children and adolescents only [6]. In 2012, another meta-analysis of GWA studies identified 2 new loci for childhood obesity in populations of European ancestry (rs9568856 of *OLFM4*, rs9299 of *HOXB5*) [5].

Asians account for 60 % of the world’s population and have higher percentages of body fat and increased metabolic disease risk than individuals of European ancestry with the same BMI. Thus, a genetic study in an Asian population can not only facilitate the dissection of genetic architecture of obesity, but also identify genetic variants of particular importance in Asians [7]. Recently, two GWA studies in East Asian populations reported 4 new loci (rs2206734 of *CDKAL1*, rs11142387 of *KLF9*, rs261967 of *PCSK1*, rs12597579 of *GP2*) associated with BMI [7, 8].

Many replication studies for the 32 loci identified by GIANT have been performed in multiple ethnic populations, including Asians [9-14]. Studies on the SNPs near *SDCCAG8* and *TNKS/MSRA* have led to mixed results [9,12,13]. Similarly, the 2 loci for childhood obesity (*OLFM4* and *HOXB5*) and the 4 loci found in East Asians (*CDKAL1*, *KLF9*, *PCSK1* and *GP2*) were not among the top hits of a large scale GWA studies meta-analysis which focused on the tails of the adult BMI distribution [13]. Recently, 28 SNPs from the 32 loci reported by GIANT and 4 additional loci identified in East Asians were studied in Chinese adults [15], but only 4 SNPs near *TMEM18*, *PCSK1*, *BDNF* and *MAP2K5* were confirmed (nominal *P*-values < 0.05). The effects of these SNPs in Chinese children were unclear.

In the present study, we genotyped 40 single nucleotide polymorphism (SNPs) and conducted association analyses of the 32 variants that had an estimated minor allele frequency >1 % in 2 030 unrelated Chinese children, including 607 normal-weight, 718 overweight, and 705 obese individuals. The purpose of this case-control study was to (a) examine whether the common variants are individually associated with obesity in Chinese children and (b) assess the cumulative effects and predictive value for obesity in Chinese children.

## Methods

### Subjects

We conducted an association study in two independent study groups, recruited from the urban regions of Beijing, China. The first study group, including 386 obese, 400 overweight and 151 normal-weight individuals, came from the study on Adolescent Lipids, Insulin Resistance and Candidate Genes (ALIR) in nine middle schools of Dongcheng District of Beijing. The second study group, including 319 obese, 318 overweight and 456 normal-weight individuals, was from the Comprehensive Prevention Project for Overweight and Obese Adolescents (CPOOA) with physical exercise and healthy nutrition as instruments in five elementary and middle schools of the Haidian District of Beijing. The ascertainment strategies for the two study groups have been described in detail previously [16,17]. The two studies were approved by the ethics committee of Peking University Health Science Center. Written informed consent was provided by all participants and, in the case of minors, their parents.

Anthropometric measurements, including height and weight, were determined according to standard protocols. BMI was calculated as weight in kilograms divided by the squared height in meters. We used the BMI percentile criteria to define obesity, overweight and normal-weight in children and adolescents, which were determined in a representative Chinese population [18]. According to the criteria (Table 1), the children and adolescents with an age- and gender-specific BMI ≥ 95^th^ percentile were defined as obese, while those with a BMI between 85^th^ and 95^th^ percentile were overweight and those with a BMI between 15^th^ and 85^th^ percentiles were normal-weight. Individuals with cardiovascular or metabolic diseases were excluded. The sex- and age-specific BMI standard deviation score (BMI-SDS) was calculated by using the growth reference data of the World Health Organization for children and adolescents aged 5–19 years [19].

**Table 1:** Body mass index reference for screening overweight and obesity in Chinese school-age children [18]

Age (years)	Boys		Girls	
	Overweight	Obesity	Overweight	Obesity
7-	17.4	19.2	17.2	18.9
8-	18.1	20.3	18.1	19.9
9-	18.9	21.4	19.0	21.0
10-	19.6	22.5	20.0	22.1
11-	20.3	23.6	21.1	23.3
12-	21.0	24.7	21.9	24.5
13-	21.9	25.7	22.6	25.6
14-	22.6	26.4	23.0	26.3
15-	23.1	26.9	23.4	26.9
16-	23.5	27.4	23.7	27.4
17-	23.8	27.8	23.8	27.7
18	24.0	28.0	24.0	28.0

The general characteristics of the study samples are shown in Table 2. The study consisted of 2 030 Chinese children, including 607 normal-weight (mean age 12.55 ± 3.04 years, mean BMI 18.77 ± 2.50 kg/m^2^), 718 overweight (mean age 13.26 ± 2.36 years, mean BMI 23.86 ± 2.20 kg/m^2^) and 705 obese (mean age 12.85 ± 2.59 years, mean BMI 28.12 ± 3.94 kg/m^2^).

**Table 2:** General characteristics of the study samples

	Normal-weight group	Overweight group	Obese group
Number	607	718	705
Female (%)	323(53.2)	268(37.3)	221(31.3)
Age	12.55 ± 3.04	13.26 ± 2.36	12.85 ± 2.59
Height(cm)	156.20 ± 15.86	161.96 ± 12.97	162.74 ± 13.81
Weight(kg)	47.09 ± 13.52	63.65 ± 13.61	76.11 ± 19.96
BMI (kg/m^2^)	18.77 ± 2.50	23.86 ± 2.20	28.12 ± 3.94
BMI-SDS	0.003 ± 0.71	1.57 ± 0.27	2.49 ± 0.48

Data are expressed as mean ± standard deviation, if not indicated otherwise. *BMI* body mass index, *SDS* Standard deviation score

### Selection of SNPs and genotyping

We selected 40 obesity-related loci identified by five recent GWA studies, with one representative SNP for each locus. Firstly, we selected the 32 SNPs reported by Speliotes et al [4]. Then we selected 4 SNPs (rs121458332 of *SDCCAG8*, rs13278851 of *TNK*S/MSRA, rs9568856 of *OLFM4*, rs9299 of *HOXB5*), which were identified by two GWA studies that focused on children [5,6]. Additionally, we selected 4 SNPs (rs2206734 of *CDKAL1*, rs11142387 of *KLF9*, rs261967 of *PCSK1*, rs12597579 of *GP2*), which were associated with BMI in two GWA studies of East Asian populations [7, 8].

Fasting venous blood samples were collected. Genomic DNA was extracted from blood leukocytes by the phenol/chloroform extraction method. Sequenom’s MassARRAY system (Sequenom, San Diego, CA, USA) was applied to genotype the 40 SNPs. Primers, including a pair of amplification primers and an extension primer for each SNP, were designed with SpectroDESIGNER software (Sequenom, San Diego, CA). A multiplex polymerase chain reaction was performed, and unincorporated double stranded nucleotide triphosphate bases were dephosphorylated with shrimp alkaline phosphatase followed by primer extension. The purified primer extension reaction was spotted onto a 384-element silicon chip (SpectroCHIP, Sequenom) and analyzed in the Matrix assisted laser desorption ionization time of flight mass Spectrometry (MALDI-TOF MS, Sequenom). The resulting spectra were processed with MassArray Typer (Sequenom, San Diego, CA).

Multiplex SNP assays designs failed for 6 out of 40 SNPs (rs10968576, rs4771122, rs10150332, rs12444979, rs29941 and rs261967); these were replaced by 6 proxy SNPs (rs16912921, rs9579083, rs10145154, rs6497416, rs29942, rs261966) with strong linkage disequilibrium (r^2^ > 0.80) in populations comparable to the discovery studies in HapMap data Release 24/phase II Nov08 (http://hapmap.ncbi.nlm.nih.gov; Han Chinese in Beijing, China (CHB) for rs261966, Utah residents with Northern and Western European ancestry (CEU) for the other 5 SNPs).

As shown in Additional file 1: Table S1, the call rates for 40 SNPs were above 95.0 %. We exclude one monomorphic (rs6497416 of *GPRC5B*), one triallelic (rs4836133 of *ZNF608*) and six rare variants with minor allele frequency below 1 % (in all genotyped individuals) from the subsequent analyses resulting in 32 SNPs. In the normal-weight group, 31 of the 32 SNPs showed no evidence for deviations from Hardy-Weinberg equilibrium (HWE; all *P* > 0.05). For one SNP (rs7138803) near *FAIM2* we observed some evidence for a deviation from HWE (*P* = 0.01) but a double-checking of the genotype data revealed no obvious genotyping artifacts.

### Statistical analyses

The genotype data of the normal-weight group were tested for deviations from Hardy–Weinberg equilibrium using χ^2^ tests (see above). *F*-statistics (*F*_ST_), a metric representing the effect of population subdivision, was calculated according to the following formula, *F*_ST_ *=* (P_1_-P_2_)^2^/ ((P_1_ + P_2_)*(2-(P_1_ + P_2_))), where P_1_ is the allele frequency estimate in the population of the discovery study and P_2_ is allele frequency estimate based on the total sample of our study [20,21]. A *F*_*ST*_ value ≥ 0.10 indicates large genetic differentiation [22].

Logistic regression was performed to examine the effect of each SNP on risk of obesity or overweight (categorical variable). Linear regression was performed to examine the effect of each SNP allele on BMI-SDS variability. Both logistic regression and linear regression were carried out under a (log)-additive genetic model with adjustment for age, age square, sex and study group (ALIR and CPOOA). For each of the 6 proxy SNPs, the allele which was correlated with the effect allele of the original SNP in the discovery study was defined as the effect allele, while the effect alleles of other SNPs were the same as the discovery studies, for comparing our results with the published data [4-8].

To identify cumulative effects of these SNPs, we created a genetic risk score (GRS) for each individual by summing up the number of effect alleles of the SNPs. We did not weight the risk alleles on the basis of their individual effect sizes because no well-accepted effect sizes were available for each of the SNPs, and it has been shown that weighting of risk alleles may have only limited effects [23]. Again logistic regression was used to calculate odds ratio (OR) of the GRS-32 from all 32 SNPs that met our minor-allele frequency cut-off (see above) for the risk of obesity or overweight. Linear regression was performed to examine the effect of GRS-32 on BMI-SDS variability.

SPSS 18.0 software was used for the above statistical analyses (SPSS, Chicago, IL). In addition to effect sizes estimates (i.e. per allele odds ratios (OR) and 95 % confidence intervals (95 % CI)), we reported nominal two-sided *P*-values. We applied a nominal significance level of α = 0.05 (two-sided). Adjustment was made for multiple testing using Bonferroni correction for 32 SNPs, i.e. resulting in α_BF_ = 0.05/32 = 0.00156 (two-sided). Difference in effect size of each SNP between our study and the discovery study was examined by testing heterogeneity with MANTRA software, which was developed by Morris AP [24] for trans-ethnic meta-analysis of genome-wide association studies. *P*(heterogeneity) is the posterior probability of heterogeneity in allelic effects, which is derived from transethnic meta-analysis. If *P*(heterogeneity) > 50 %, there is the evidence of heterogeneity in allelic effects between the present and discovery studies [24]. The receiver operating characteristic (ROC) curves comparing normal-weight and obese children were produced by logistic regression, and the areas under the curve (AUC_ROC_) from different models were compared by MedCalc software. Based on the published minor allele frequencies (see Table 3) and applying a (log)-additive genetic model, a sample size of 705 obese cases and 607 normal-weight controls has a comparisons-wise power ranging between 0.94-0.99 for a true allelic OR of 1.5 or 0.33-0.64 for a true allelic OR of 1.2 (α = 0.05; two-sided). Accounting for multiplicity these numbers changed to 0.63-0.98 or 0.05-0.20, respectively (α_BF_ = 0.00156; two-sided). Similarly, analyzing BMI-SDS in a sample of 2 030 children, leads to a comparisons-wise power ranging between 0.51-0.89 for a true allelic β of 0.10 (in units of BMI-SDS) or 0.17-0.36 for a true allelic β of 0.05 (α = 0.05; two-sided). Accounting for multiplicity these numbers changed to 0.12-0.51 or 0.02-0.06, respectively (α_BF_ = 0.00156; two-sided). These power calculations were performed using Quanto software (University of Southern California, Los Angeles, CA).

**Table 3:** Hardy-Weinberg equilibrium (HWE) test and effect allele frequency (EAF) in Chinese children for32 GWA studies -derived SNPs

SNP	Nearest gene	Chr	Position	Allele		HWE *P*-value^a^	EAF^b^	EAF^c^	*F*_ST_
				Effect	Other				
Speliotes et al. 2010 (in children and adults of European ancestry) [4]									
rs1558902	*FTO*	16	52361075	A	T	0.58	0.13	0.42	0.105^e^
rs2867125	*TMEM18*	2	612827	C	T	0.20	0.91	0.83	0.014
rs571312	*MC4R*	18	55990749	A	C	0.67	0.23	0.24	0.000
rs10938397	*GNPDA2*	4	44877284	G	A	0.54	0.31	0.43	0.015
rs10767664	*BDNF*	11	27682562	A	T	0.30	0.55	0.78	0.059
rs2815752	*NEGR1*	1	72585028	A	G	0.97	0.92	0.61	0.134^e^
rs7359397	*SH2B1*	16	28793160	T	C	0.17	0.16	0.40	0.071
rs9816226	*ETV5*	3	187317193	T	A	0.36	0.97	0.82	0.060
rs3817334	*MTCH2*	11	47607569	T	C	0.16	0.33	0.41	0.007
rs29942^d^	*KCTD15*	19	39001117	C	T	0.76	0.25	0.31	0.004
rs543874	*SEC16B*	1	176156103	G	A	0.80	0.22	0.19	0.001
rs987237	*TFAP2B*	6	50911009	G	A	0.52	0.18	0.18	0.000
rs7138803	*FAIM2*	12	48533735	A	G	0.01	0.28	0.38	0.011
rs713586	RBJ	2	25011512	C	T	0.77	0.49	0.47	0.000
rs2241423	*MAP2K5*	15	65873892	G	A	0.67	0.41	0.78	0.142^e^
rs2287019	*QPCTL*	19	50894012	C	T	0.09	0.82	0.80	0.001
rs1514175	*TNNI3K*	1	74764232	A	G	0.91	0.78	0.43	0.128^e^
rs2112347	*FLJ35779*	5	75050998	T	G	0.88	0.44	0.63	0.036
rs16912921^d^	*LRRN6C*	9	28403461	A	C	0.19	0.30	0.34	0.002
rs3810291	*TMEM160*	19	52260843	A	G	0.58	0.29	0.67	0.145^e^
rs1555543	*PTBP2*	1	96717385	C	A	0.81	0.87	0.59	0.099
rs9579083^d^	*MTIF3*	13	26915270	C	G	0.87	0.15	0.23	0.010
rs4929949	*RPL27A*	11	8561169	C	T	0.79	0.40	0.52	0.014
rs206936	*NUDT3*	6	34410847	G	A	0.30	0.51	0.21	0.098
Scherag et al. 2010 (in children and adults of European ancestry) [6]									
rs12145833	*SDCCAG8*	1	241550377	T	G	0.55	0.91	0.87	0.004
rs13278851	*TNKS/MSRA*	8	9788282	A	G	0.28	0.14	0.11	0.002
Bradfield et al. 2012 (in children of European ancestry) [5]									
rs9568856	*OLFM4*	13	52962982	A	G	0.34	0.33	0.16	0.039
rs9299	*HOXB5*	17	44024429	A	G	0.44	0.53	0.65	0.015
Okada et al. 2012 (in East Asians) [7]									
rs2206734	*CDKAL1*	6	20802863	G	A	0.66	0.59	0.59	0.000
rs11142387	*KLF9*	9	72188152	C	A	0.96	0.33	0.46	0.018
Wen et al. 2012 (in East Asians) [8]									
rs261966^d^	*PCSK1*	5	95875343	G	A	0.53	0.45	0.42	0.001
rs12597579	*GP2*	16	20165368	C	T	0.99	0.73	0.80	0.007

*Chr* Chromosome; Position: NCBI build 36.3 (NCBI, Bethesda, MD); *EAF* Effect allele frequency, *HWE* Hardy-Weinberg equilibrium

^a^HWE *P*-value in normal-weight Chinese children

^b^Effect allele frequency in all genotyped individuals of the present study

^c^Effect allele frequency of SNPs in the discovery studies (Ref. [4-8]), and that of each proxy SNP is from NCBI database among the same populations that in the discovery study

^d^Proxy SNPs were used in the present study to replace the SNPs from the discovery studies

^e^ *F*_ST_ > 0.10 as indicator of large genetic differentiation between population in the present study and that in the discovery study

## Results

### Effect allele frequencies

The effect allele frequencies of 32 SNPs and *F*_ST_ values between the population in the present study and that in the discovery study are shown in Table 3. All effect allele frequencies in the present study were similar to those reported in the HapMap Han Chinese (http://hapmap.ncbi.nlm.nih.gov/). The *F*_ST_ values between the present study and the discovery study varied from 0 (rs987237 of *TFAP2B*, rs2206734 of *CDKAL1*) to 0.145 (rs3810291 of *TMEM160*). Based on the *F*_ST_ values, we found that 23/28 SNPs from three GWA studies of Europeans and 4/4 SNPs from two GWA studies of East Asians had similar effect allele frequencies in our study. Only 5 SNPs near *FTO, MAP2K5, NEGR1, TNN13K, TMEM160* from the GIANT study on BMI variability showed large genetic differentiation between the Europeans and our study population (*F*_*ST*_ value ≥ 0.10).

### Individual associations of 32 SNPs with obesity

**Fig. 1:**
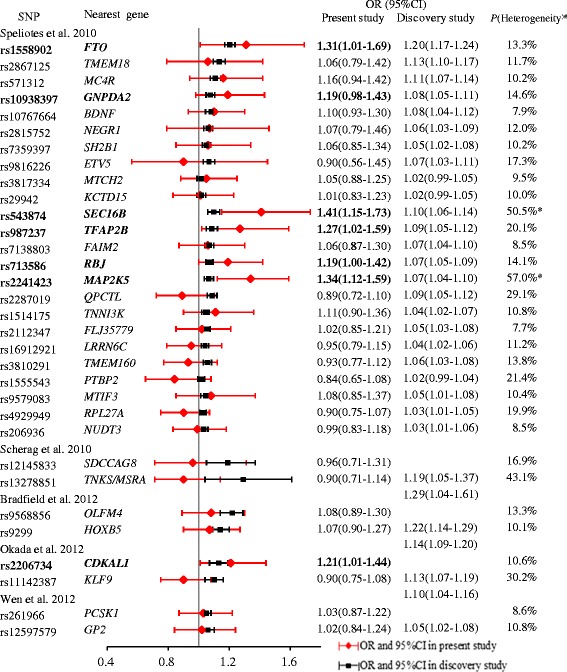
Forest plot showing the ORs (95 % CI) for associations between obesity and 32 SNPs in this study and the reported ORs in the discovery GWA studies. SNP alleles which are associated with obesity risk in Chinese children are highlighted in bold. ^#^ *P*(heterogeneity) is the posterior probability of heterogeneity in allelic effects, which is derived from transethnic meta-analysis [24] *P(heterogeneity) > 50 %, providing evidence of heterogeneity in allelic effects between the present and discovery studies

Table 4 shows the results of the allelic association analyses of the 32 SNPs with obesity in Chinese children. We identified the nominally significant associations with obesity for effect alleles of 6 SNPs at *FTO, SEC16B, TFAP2B, RBJ, MAP2K5* and *CDKAL1* (ORs for the effect allele ranged between 1.19 and 1.41, nominal two-sided *P* < 0.05). SNP rs543874 near *SEC16B* and rs2241423 near *MAP2K5* remained significant after Bonferroni correction for multiple testing (*P* < 0.00156, Bonferroni corrected for 32 SNPs). Fig. 1 shows ORs and 95 % CI for the association with obesity for the 32 SNPs in the present study and the published ORs for each SNP reported in the GWA studies. As shown in Fig. 1, overall 30 of the 32 SNPs yielded directionally consistent effects, i.e. the ORs of the SNPs in this study were comparable with those detected in the discovery studies. However, the effect sizes of two SNPs at *SEC16B* and *MAP2K5* showed heterogeneity between the present and discovery studies (*P*(heterogeneity) > 50 %). The associations of obesity with SNP alleles at rs543874 of *SEC16B* (OR = 1.41, 95 % CI: 1.15-1.73) or at rs2241423 of *MAP2K5* (OR = 1.34, 95 % CI: 1.12-1.59) seemed to be stronger in Chinese children than in Caucasians (OR = 1.10, 95 % CI: 1.06-1.14; OR = 1.07, 95 % CI: 1.04-1.10, respectively) [4], which was also indicated by non-overlapping 95 % confidence intervals in Fig. 1.

**Table 4:** Association of 32 GWA studies-derived SNP alleles with obesity in Chinese children

SNP	Nearest gene	Chr.	Allele		Obesity VS Normal-weight		
			Effect	other	OR	95 % CI	Two-sided
							*P*-value
Speliotes et al. 2010 (in children and adults of European ancestry) [4]							
**rs1558902**	***FTO***	**16**	**A**	**T**	**1.31**	**1.01-1.69**	**0.039**
rs2867125	*TMEM18*	2	C	T	1.06	0.79-1.42	0.703
rs571312	*MC4R*	18	A	C	1.16	0.94-1.42	0.166
rs10938397	*GNPDA2*	4	G	A	1.19	0.98-1.43	0.077
rs10767664	*BDNF*	11	A	T	1.10	0.93-1.30	0.273
rs2815752	*NEGR1*	1	A	G	1.07	0.79-1.46	0.645
rs7359397	*SH2B1*	16	T	C	1.06	0.85-1.34	0.597
rs9816226	*ETV5*	3	T	A	0.90	0.56-1.45	0.669
rs3817334	*MTCH2*	11	T	C	1.05	0.88-1.25	0.626
rs29942	*KCTD15*	19	C	T	1.01	0.83-1.23	0.949
**rs543874**	***SEC16B***	**1**	**G**	**A**	**1.41**	**1.15-1.73**	**0.001**
**rs987237**	***TFAP2B***	**6**	**G**	**A**	**1.27**	**1.02-1.59**	**0.032**
rs7138803	*FAIM2*	12	A	G	1.06	0.87-1.30	0.542
**rs713586**	***RBJ***	**2**	**C**	**T**	**1.19**	**1.00-1.42**	**0.048**
**rs2241423**	***MAP2K5***	**15**	**G**	**A**	**1.34**	**1.12-1.59**	**0.001**
rs2287019	*QPCTL*	19	C	T	0.89	0.72-1.10	0.283
rs1514175	*TNNI3K*	1	A	G	1.11	0.90-1.36	0.332
rs2112347	*FLJ35779*	5	T	G	1.02	0.85-1.21	0.865
rs16912921	*LRRN6C*	9	A	C	0.95	0.79-1.15	0.628
rs3810291	*TMEM160*	19	A	G	0.93	0.77-1.12	0.456
rs1555543	*PTBP2*	1	C	A	0.84	0.65-1.08	0.174
rs9579083	*MTIF3*	13	C	G	1.08	0.85-1.37	0.519
rs4929949	*RPL27A*	11	C	T	0.90	0.75-1.07	0.215
rs206936	*NUDT3*	6	G	A	0.99	0.83-1.18	0.919
Scherag et al. 2010 (in children and adults of European ancestry)[6]							
rs12145833	*SDCCAG8*	1	T	G	0.96	0.71-1.31	0.799
rs13278851	*TNKS/MSRA*	8	A	G	0.90	0.71-1.14	0.375
Bradfield et al. 2012 (in children of European ancestry) [5]							
rs9568856	*OLFM4*	13	A	G	1.08	0.89-1.30	0.438
rs9299	*HOXB5*	17	A	G	1.07	0.90-1.27	0.449
Okada et al. 2012 (in East Asians) [8]							
**rs2206734**	***CDKAL1***	**6**	**G**	**A**	**1.21**	**1.01-1.44**	**0.035**
rs11142387	*KLF9*	9	C	A	0.90	0.75-1.08	0.268
Wen et al. 2012 (in East Asians) [7]							
rs261966	*PCSK1*	5	G	A	1.03	0.87-1.22	0.764
rs12597579	*GP2*	16	C	T	1.02	0.84-1.24	0.871

SNP alleles which are associated with obesity risk in Chinese children are highlighted in bold. *Chr.* Chromosome

We also found directionally consistent associations of the 6 SNPs with risk of overweight (see Additional file 2: Table S2), but none was significant after Bonferroni correction for 32 loci.

### Individual association of 32 SNPs with BMI-SDS

We additionally examined the association between the 32 common variants and BMI-SDS variability in all 2 030 children (see Additional file 3: Table S3). There were 5 SNPs that showed significant evidence for allelic association with BMI-SDS variability (nominal *P* <0.05); except for rs2206734 (*P* = 0.133), 5 of the 6 SNPs associated with obesity were also associated with BMI-SDS. Again the allelic association of rs543874 near *SEC16B* and rs2241423 near *MAP2K5* remained significant after Bonferroni correction for multiple testing (*P* < 0.00156, Bonferroni corrected for 32 SNPs).

### Cumulative effects of these SNP alleles

**Fig. 2:**
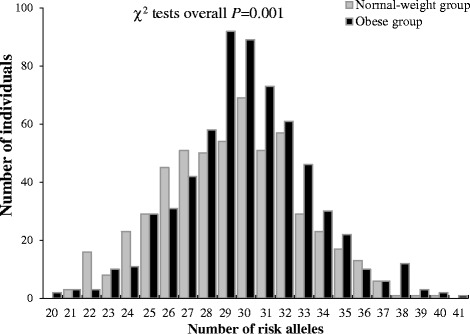
Distributions (histograms) of the number of risk alleles at 32 loci from GWA studies in obese (black) and normal-weight (gray) Chinese children.

Figure 2 shows a shift towards an increased number of risk alleles in the obese group compared to the controls (χ^2^ tests overall *P* = 0.001). To assess the cumulative effects of 32 SNPs, we created a GRS-32 by summing up the number of effect alleles of the SNPs. Multiple logistic regression revealed that on average, each additional effect allele of GRS-32 was associated with an adjusted 1.06-fold increased odds for obesity (95 % CI: 1.03-1.11, *P* = 4.84 × 10^-4^). Similarly, the average per allele increase for BMI-SDS was 0.04 (95 % CI: 0.02-0.06, *P* = 3.69 × 10^-7^) in the overall sample. The 32 variants explained 3.30 % of the variance in BMI-SDS.

The area under the ROC curve (AUC_ROC_) for prediction of risk of obesity using a model including only covariates (age, age square, sex and study group) was 0.735 (95 % CI: 0.709-0.760), which increased to 0.763 (95 % CI: 0.738-0.787) when additionally including the GRS-32 (Difference: 2.8 %, *P* = 0.0002 for difference between the two models).

## Discussion

In this cross-sectional study, we investigated the association of 32 common variants identified by five recent GWA studies in Chinese children. Except for a recent study of 28 SNPs in Chinese adults [15], only 12 loci identified in European population prior to 2010 have been investigated for their effects on BMI or obesity in Chinese populations so far [14, 25-29].

We found nominally significant associations to obesity risk in Chinese children for effect alleles of 6 SNPs near *FTO, SEC16B, TFAP2B, RBJ, MAP2K5* and *CDKAL1*. We compared our findings to the results of a recent study among Chinese adults aged 50-70 years [15]. Although the 6 nominally significant SNP alleles of our study were not significant in that study, the 95 % CIs of ORs overlapped. Similarly, some SNP alleles identified by recent GWA studies [4-8] also confer susceptibility to obesity to Chinese though not meeting a formal significance level. Further exploration of our data revealed that the SNP alleles near *SEC16B* and *MAP2K5* had presumably even stronger effects on obesity in Chinese children than in Caucasian populations (as based on the non-overlap of confidence intervals) and remained significant after correcting for multiple testing. These findings imply possible ethnic differences for effect sizes which have not been reported previously. Large-scaled studies or meta-analyses are required to clarify the ethnic difference of effect size.

In search of a better understanding of the genetic etiology of obesity and given the small individual effect sizes for loci identified in GWA studies that are likely missed applying formal significance testing, many researchers have aggregated information across loci to calculate a genetic risk score from the sum of risk alleles accumulated in an individual [30]. We calculated a genetic risk score for the cumulative effects of all 32 common variants from five GWA studies in Chinese children, including 4 SNPs associated with childhood obesity and 4 SNPs identified in East Asians. We showed that these variants had cumulative effects but a limited predictive value for obesity, which is consistent with previous studies in different populations [2, 14, 31-34].

There are several possible explanations for confirmation of only 6 SNPs in our sample. Firstly, the true effects of the 26 SNPs without formal statistical significance might be smaller than in original populations – a phenomenon called the winners curse. Consequently, our study would be underpowered to detect the effects. We noted only one of the four SNPs (rs2206734 of *CDKAL1*) that were initially associated with in East Asians achieved significance in this study. However, all these 26 SNPs without statistical significance (including 3 SNPs of East Asians) had directionally consistent effects on obesity compared to the original studies (Fig. 1). Moreover, the cumulative effect analysis of all 32 SNPs demonstrated a clear dosage effect, suggesting polygenic contribution of the alleles at these loci with smaller effect sizes. Secondly, our data suggest that there is a possible ethnic differentiation between Chinese and other ethnic groups. Among 26 loci without a formal significant association, 3 loci (*NEGR1, TNN13K, TMEM160*) had different effect allele frequencies between Europeans and our Chinese individuals (*F*_*ST*_ value ≥ 0.10). Thirdly, none of the 6 proxy SNPs showed significant association, which awaits further studies in difference of linkage disequilibrium between Chinese and Caucasian populations.

The strengths of our study include: (a) Anthropometric measurements were taken by trained interviewers according to a standard protocol which minimized measurement errors; (b) Our study groups were relatively homogeneous, both coming from the urban area of Beijing; (c) Our study was conducted in Chinese children. Compared with adults, children have higher BMI or obesity heritability and most obese children have simple obesity without complications, which help to identify the effects of common variants on obesity.

The main limitation of the present study is the relatively small sample size and consequently, reduced statistical power. Moreover, the study is limited by the number of loci tested, which is growing as new genome-wide meta-analyses are conducted [35].

## Conclusion

In conclusion, 6/32 tested SNPs were individually associated with obesity in Chinese children. Particularly the effect alleles at two SNPs near *SEC16B* and *MAP2K5* had presumably stronger effects in Chinese children than in Caucasian populations. The 32 common variants identified by recent GWA studies had cumulative effects and predictive value for obesity in Chinese children. The study demonstrated the value of conducting genetic studies in different ethnic populations. Assuming that the high heritability estimates for obesity are correct, these results suggest that more common variants, along with rare variants, copy number variants, epigenetic effects or gene-gene and gene-environment interaction remain to be identified.

## Additional files

Additional file 1: Table S1. Basic genotyping information of 40 GWA studies-derived SNPs in Chinese children. (XLSX 16 kb) Additional file 2: Table S2. Association of 32 GWA studies-derived SNP alleles with overweight in Chinese children. (XLSX 13 kb) Additional file 3: Table S3. Association of 32 GWA studies-derived SNP alleles with BMI standard deviation score (BMI-SDS) variability in Chinese children. (XLSX 13 kb)

## Abbreviations

GWA: Genome-wide association
BMI: Body mass index
SNPs: Single nucleotide polymorphisms
AUC_ROC_: Area under the receiver operating characteristic curve
ORs: Odds ratios
BMI-SDS: BMI standard deviation score
GIANT: Genetic Investigation of Anthropometric Traits consortium
ALIR: Study on Adolescent Lipids, Insulin Resistance and Candidate Genes
CPOOA: The Comprehensive Prevention Project for Overweight and Obese Adolescents
*F*_ST_: *F*-statistics
GRS: Genetic risk score
ROC: Receiver operating characteristic curves.
